# Understanding the information-seeking behavior of pharmacy college faculty, staff, and students: implications for improving embedded librarian services

**DOI:** 10.5195/jmla.2021.950

**Published:** 2021-04-01

**Authors:** Hitoshi Kamada, Jennifer R. Martin, Marion K. Slack, Sandra S. Kramer

**Affiliations:** 1 hkamada@notredame.ac.jp, Associate Professor, Department of Japanese and Global Cultures, Kyoto Notre Dame University, Japan; 2 jenmartin@.arizona.edu, Librarian, Research & Learning, Arizona Health Sciences Library, and Clinical Instructor, Pharmacy Practice and Science, College of Pharmacy, The University of Arizona, Tucson, AZ; 3 slack@pharmacy.arizona.edu, Professor Emeritus, Pharmacy Practice and Science, College of Pharmacy, The University of Arizona, Tucson, AZ; 4 skramer@email.arizona.edu, Associate Librarian, (Retired), Research & Learning, Arizona Health Sciences Library, The University of Arizona, Tucson, AZ

**Keywords:** information-seeking behavior, embedded librarian, pharmacy education

## Abstract

**Objective:**

Research was conducted on the embedded librarian program at The University of Arizona College of Pharmacy and the Health Sciences Library to understand how this service is relevant to users and identify the potential for further improvement. This study examined users’ information-seeking behaviors and considered the implications for the effectiveness of the embedded librarian service.

**Methods:**

The authors conducted 18 semi-structured interviews of faculty, researchers, and students at the College of Pharmacy to obtain descriptive accounts of how they seek information, manage information, and use the library and library services. The authors examined the interview transcripts through qualitative descriptive analysis.

**Results:**

The interview responses confirm that users seek information outside of the physical library and tend to ask their peers for information or assistance in obtaining information. They mostly feel comfortable in searching, but some of them may lack sufficient search skills and tend to use a few known databases. While those who are familiar with the librarian seek the librarian's assistance more often, others tend not to seek the librarian's assistance. The ways they manage information vary, which requires customized assistance.

**Conclusions:**

The close proximity of a physically embedded librarian is beneficial to users and positions the librarian to provide proactive assistance in the existing user information-seeking behavior environment. While some users do not seek assistance, the embedded librarian can provide proactive assistance in such areas as making users aware of other database options and helping them choose relevant databases and effectively manage information.

## INTRODUCTION

The environment surrounding health sciences information and libraries is changing rapidly. Almost all information resources are now online, and most users now discover and utilize these resources outside a physical library space [1]. Health sciences libraries at higher education institutions have responded to these trends in a variety of ways. As one example, the University of Arizona Health Sciences Library (UAHSL) initiated an “embedded librarians” approach in 2005 whereby librarians are housed within the health sciences colleges and provide information services primarily to users within these colleges [2]. While the librarian is physically embedded in the college, an effective embedded librarian is not just physically embedded within an academic unit but is also a part of users’ information activities. To work toward this, librarians must better understand users’ information needs and information-seeking behaviors. This study examines users’ information-seeking behaviors in detail and considers how the embedded model can have the greatest impact on their information-seeking and use.

Within the library profession, the term “embedded librarians” has been used to describe different activities in various settings. Wu and Mi outlined models for embedded librarians in health sciences libraries, in which they articulated roles and duties performed by embedded librarians [3]. Wu and Thornton also found that the embedded model plays a key role in delivering information at the point of need in pharmacy curriculum. In addition, building relationships in the college provides value as the embedded librarian becomes a visible model of support [4]. Blake et al. conducted a survey of health sciences departments and found that the mere presence of a librarian in a department might encourage users to use the librarian's service more frequently, although awareness of the librarian's services was low among users [5]. Kumar et al. conducted surveys before and after an online course on health informatics graduate research that involved an embedded librarian and found that students’ confidence in their research skills increased after the course was completed [6]. Furthermore, Wu et al. surveyed students to evaluate an embedded librarian project for an online nursing course and found that the librarian's services were effective at point of need for students in the course [7]. Thus, the effectiveness of the embedded librarian model is supported by various measures throughout the literature.

Research on information needs and information-seeking behavior in health sciences usually focuses on particular disciplines. Lê conducted a survey of public health students and discussed how they might not be fully aware of subject-specific resources, particularly a diverse range of resources about which the librarian can provide targeted information and instruction [8]. A survey of medical students found that they must go through a large amount of information to locate specific information and resources applicable in the clinical setting [9]. Interviews with basic science researchers at a medical school revealed that researchers tend to use resources and channels other than librarians to obtain information [10]. In another study, the use of online resources by health sciences faculty showed that MEDLINE was a popular place to start searching [11]. The use of Google or Google Scholar as well as library databases for information seeking is one common thread found in this literature [8–11]. A survey of health sciences faculty affirmed their reliance on journals and electronic resources as sources of information, with additional findings confirming the importance of current journals for keeping users up to date and faculty's preference for individual librarian assistance rather than in-class instruction [1]. Twiss-Brooks et al. conducted ethnographic studies of the information-seeking behavior of third-year medical students and examined their use of information resources, technology, and spaces [12]. Their findings identified convenience and recommendations from others as influential factors in medical students’ choice of information resources. All of these studies investigated patterns of user information-seeking behavior leading to improvements and targeted information services and instruction by a librarian.

A previous study at the UAHSL used focus groups to understand the information needs of pharmacy faculty and obtain their feedback about the librarian liaison program [13]. However, additional studies that include different types of users, such as graduate students, could help libraries better support a wider range of users within colleges of pharmacy. While overall trends in information-seeking behavior can be identified using surveys, we chose to conduct interviews to obtain individual accounts of local users’ information-seeking behavior to improve our embedded librarian services at the UAHSL. Thus, this study also adds to the literature by studying how embedded librarian services are used through qualitative examination of users’ information-seeking behavior. The objectives of this study are as follows: (1) to understand local users’ information-seeking behavior by obtaining detailed and specific accounts of when and how individuals seek and manage information and how they feel during these activities; and (2) to identify where and how the embedded librarian service may be positioned effectively within users’ information-seeking processes.

## METHODS

At the University of Arizona, the office of the embedded pharmacy liaison librarian is physically located in the building where the College of Pharmacy faculty, staff, and students work and where onsite library services are provided. The University of Arizona is one of the first colleges that implemented the embedded liaison model. The pharmacy librarian integrates library instruction within the Doctor of Pharmacy (PharmD) didactic curriculum, provides research support for graduate students, and collaborates and supports faculty research and scholarship.

### Interview design

One-on-one semi-structured interviews were used to capture personal accounts of how users seek and use information. We developed the interview questions with reference to the literature on information-seeking behavior in the health sciences setting and specific institutional needs at the UAHSL. This study was approved by the Human Subjects Protection Program Institutional Review Board (IRB) at the University of Arizona.

The interview questions were pre-tested with three individuals at the College of Pharmacy who did not formally participate in the study to ensure that the questions would make sense to interview participants and solicit relevant responses. A prepared set of questions was asked, with follow-up questions added as needed (Appendix A). The first half of the interview asked participants about a recent experience when they needed to seek information for research or educational purposes.?Participants were also asked to describe how they decided which information resource to use, how they performed their search, and how they determined when they could conclude their information search process. The interviews attempted to capture participants’ satisfaction or dissatisfaction with their search results and any difficulties they encountered during their search process. The second half of the interview asked about participants’ general search behavior outside of specific information-seeking activities, such as how often information was sought and if they used citation management software. The last part of the interview asked how participants felt about using the library and contacting a librarian for assistance. Participants were also asked about their current and previous experience using the library and receiving library instruction or orientation.

### Data collection

To recruit participants, email invitations were sent to all of the approximately 400 College of Pharmacy faculty, staff, and students. Gift cards were offered to solicit participation. Notwithstanding this incentive, the invitation may have attracted those who had a stronger interest in library services than those who did not respond. Participants were asked to fill out an online form to collect demographic information before participating. Participants completed the in-person interviews individually. At least three investigators were present at each interview. Some of the investigators who conducted the interviews were librarians, so participants’ responses may have been biased toward expressing a positive attitude towards library services. We were mindful of this potential source of bias and made efforts to solicit candid responses. The interviews ranged from 25 minutes to one hour and 20 minutes, depending on the length of participants’ responses.

### Data analysis

Each interview was recorded and transcribed. The transcripts for each interview were analyzed qualitatively with NVivo 11 (QSR International) analysis software. Initial codes were assigned to help organize the transcribed data by topic, and subsequent codes that emerged from the data were assigned to identify major themes. When assigning codes and making annotations, data were examined individually and across participants to identify similarities and differences among responses. Transcriptions were reexamined continuously during the period of analysis. In addition to online communication and data sharing, we met several times to discuss the coded data. Through this process, we attempted to ensure that?the analysis and results accurately portrayed the original data.

### Results

A total of 18 people from the College of Pharmacy participated in an interview. These participants consisted of three faculty members (17%), three professional staff (17%), three PhD/MA students (17%), eight students in the PharmD program (44%), and one undergraduate student (5.5%) (Table 1). The interviews took place over a period of four months.

**Table 1:** Participant Demographics^*^

Demographic	Number of Participants
**Gender**	
Women	9 (50%)
Men	9 (50%)
**Classification**	
Faculty	3 (17%)
Staff	3 (17%)
P1	4 (22%)
P2	1 (5.5%)
P3	2 (11%)
P4	1 (5.5%)
Graduate Student	3 (17%)
Undergraduate Student	1 (5.5%)

* P1: First professional pharmacy year; P2: Second professional pharmacy year; P3: Third professional pharmacy year; P4: Fourth professional pharmacy year

Findings from the interviews are summarized in Table 2.

**Table 2:** Summary of Key Findings and Example Quotes

Key Findings	Example Quotes
Users tend to use a few known databases for initial information searching, and their awareness of other database options varies.	“There are a ton of databases that are available to me. Those happen to be the two that happen to be the most pertinent to what I do, mainly chemical synthesis.” (Participant 28) “I used Embase a few times, but I am unfamiliar with them as compared to the PubMed stuff. PubMed is what I always fall back to.” (Participant 12)
Users are mostly confident in searching, including coming up with keywords.	“Usually I will get adequate results but sometimes I will run into a little trouble finding primary literature that matches what I am looking for but the majority of the time I do get what I am looking for.” (Participant 18) “I know there are sophisticated ways with Mesh terms but for me I am satisfied with running a search with a keyword, running another search with another key word and then just starting to combine search terms at that point. Then, winnowing down my search hits until I get what I want.” (Participant 24)
Users devise their own ways of managing information and keeping up to date. They may be receiving too much information or are not sure what information is relevant.	“I used RefWorks one time to help with the citations but just the way the teachers want the specific format, I will just end up doing it on my own. That's easier rather than having to go through the whole process of specific format.” (Participant 18) “Typically, what I will do is I will download the papers that I am looking for and I use Mendeley to organize them so I have a folder for each of my projects and I keep things organized that way. That's a really nice way to keep track of everything.” (Participant 28)
Users say they are comfortable using library services and are willing to ask a librarian, but they do not necessarily do so.	“I’m usually okay with handling searches like this by myself. I’ve never really needed to seek out professional help with it.” (Participant 7) “I guess if it were a technical problem then I might ask for help. I don’t usually think to ask the librarian for help.” (Participant 14)
When users become familiar with a librarian, they utilize that librarian's service more often.	“Our librarian here is very responsive and available and accessible to us any time I have ever had to refer to her. No. It is excellent resource.” (Participant 24) “So I have come to Jennifer for some assistance in the middle of writing a review article on FDA approved biologics drugs. […] But when I have needed sort of more sophisticated search tools I have actually met with Jennifer before and she showed me some of these tools. (Participant 28)

## SEEKING AND USING INFORMATION

### Seeking information

Seven participants stated they retrieved information daily; seven others sought information once or several times a week. When participants began searching for information, they tended to use one or a few databases with which they were familiar. Except for one student, all participants mentioned the use of PubMed. Specialized subject-specific databases, such as Embase, were also noted. The degree of awareness of other databases differed among participants. Experienced searchers used their favorite databases but were aware of other database options. For example, a student in the PharmD program stated:

In terms of finding, it is not very confusing to find any information as long as I know the keywords to find the information and know what sources to use. I always go to the Arizona Health Sciences Library website and there are all the databases and I just look through them. (Participant 9)

In addition, participants articulated the importance of context when conducting a search and would look for background information before initiating a more detailed search. For example, a non-faculty researcher stated:

You should go in knowing what type of topic you are looking for. If you don’t know the keywords, then maybe you should do more research to find out what is specific for what you are looking for and to help separate it out from other things that may be too similar. (Participant 23)

While they were aware of databases other than their preferred databases, they sometimes felt overwhelmed by the number of databases available. The same participant also stated, “I would probably ask for help because I know there is a lot of other databases out there so I wouldn’t be sure what website would be best to search for what I was looking for.” (Participant 23)

On the other hand, there were several occasions when participants would only search one database that they were familiar with. For example, one PharmD student stated: “Embase was my only source. Embase is the only one I am comfortable using.” (Participant 21) Also, their initial starting point may not have been appropriate. One participant expressed difficulty in searching and started searching electronic journals directly before using a relevant database:

Interviewer: Did you think of other sources you might have searched?

Not really. Most of the time I’ll do just the general health sciences library journal search. If that doesn’t come up with anything, then I’ll go into specific databases. (Participant 7)

The responses from the interview sessions generally showed combined use of Google and other subject databases, not a sole reliance on Google or Google Scholar. For example, one PharmD student talked about the combined use of Google and PubMed:

If I’m having trouble finding things on PubMed I might just go to Google or Google Scholar specifically because that's going to give me even more options, but I try to avoid that until I really can’t, until I can’t find exactly what I am looking for, what I want to find. (Participant 14)

The majority of participants stated they did not have much difficulty coming up with keywords. This was especially the case when they were familiar with the topic or their information needs were straightforward. In some instances, they searched Google for keywords. Eight participants mentioned using controlled vocabularies such as the medical subject heading (MeSH) in PubMed. For example, when asked to describe how they came up with keywords for a search, one Pharm D student stated:

It is pretty easy, especially with the MeSH terms. You have to be careful about which ones you pick so you don’t exclude stuff that does apply to your research that you are going to miss. That is why Google is a backup to catch stuff that is kind of similar. Because it gives less exact results than using a MeSH term. (Participant 12)

In addition, the following comments mention the use of related terms generated automatically by the database:

Coming up with keywords. That's not too difficult, especially in Emtree because it will generate other terms. (Participant 17)

The same thing with Embase, where you type in a keyword and it will give you what is most commonly used right there and you can input it right there. (Participant 18)

Participants generally expressed satisfaction with their search results. For example, one participant stated, “Typically I am pretty confident in the results that I get. They usually lead me toward the answers that I was looking for. I imagine they are good.” (Participant 23) When they could not find what they were looking for, they found it difficult. “Not great. Like I said, I only found that one article and I’d like to be able to cite more.” (Participant 7) They occasionally felt difficulty when encountering a large search result. One student talked about difficulty narrowing down results when searching Google Scholar:

It is sometimes a little hard to do the narrowing down by academic area. Sometimes I don’t have the right strategy to do it. That's something I could search through individual journals for, as opposed to Google Scholar or things like that. Once I find one article of a specific journal in the subject I’m looking for, I can go that route. But if I am looking for doing it in a global search, then it's hard to narrow it down from there. (Participant 8)

Participants’ responses suggested that confidence in the search results may not always correlate with one's search skills. For example, one PharmD student was satisfied with their results even though, in one instance, this student had conducted only one search in Google to locate published guidelines. Health information databases are more appropriate for locating this type of information. Further, participants’ confidence also depends on the degree of one's expectations and requirements for needed information. For example, one faculty member stated:

You think that there's more information. You’re not sure that you’re good enough, having resources to be able to do an actually stellar search and know that then you’ve got everything that's out there. It just changes how you write. (Participant 22)

As people become accustomed to being able to obtain more information online, they find the non-electronic availability of some information frustrating. This is not only the case for “digital native” students, but also for faculty members who work in a clinical setting where obtaining information is time sensitive for patient care. The same faculty member stated: “When I can’t just click on the article and have access to it immediately, then it's really frustrating.” (Participant 22)

When conducting exhaustive searches, such as for a systematic review, deciding when to stop searching becomes a difficult task. For example, a researcher said:

That's sometimes tricky because sometimes there's always more that you can find on a topic. So maybe in my experience, I kind of figure out as much as I can and when I start seeing things that are repetitive or things that I am reading that I’ve already read somewhere else or I get to the point where I am just seeing things again and again, then I usually assume that I probably figured out as much as I can at that point about what I am looking for. (Participant 23)

## MANAGING INFORMATION

Methods of managing information for research and study varied widely among participants. About half of them had used citation management software such as RefWorks, Mendeley, and EndNote. Other methods of managing information included copying the URLs of articles, pasting bibliographic information into a Word document, bookmarking articles in a browser, saving bibliographic information in email, and saving DOIs of the articles. While they followed article references, they often did not use citation databases, such as Web of Science, to identify new articles. One-third of participants, mainly faculty, staff, and PhD students, mentioned the use of these databases for citation searches. However, they did not indicate frequent use of these databases.

Participants’ responses to the question of how they keep up to date with recent research also varied widely. They received current information through newsletters and mailing lists; they also got current information from personal contacts. All three faculty members mentioned that they receive the table of contents of journals via email, while students did this less often. Participants’ responses revealed that they felt they received too much information or received it too often. One PharmD student expressed frustration: “A lot of times emails come from them and it is a little overwhelming. I do not look at them.” (Participant 10) In addition, a couple of students did not see enough relevance in the information they received. One PharmD student stated, “I think we get a free subscription to the Pharmacist Letter. I haven’t exactly utilized that, though.” (Participant 17) Another PharmD student stated:

I do receive updates of pharmacy information, but usually I don’t explore those very much just ‘cause at this point I don’t really know how to apply that and I don’t know when it's going to change because I am not really practicing yet. I don’t always feel like it's the most relevant, so I don’t explore those too much. But I do receive them. (Participant 14)

Participants also turned to peers for information. For example, a PharmD student stated:

If I am having trouble finding a source for a question, then I will ask other people about it. Most cases, I will ask the students in my class, not outside of class. Because we are in the same boat usually. (Participant 9)

The same student also said that when in the laboratory setting, they would ask their supervisor or a senior graduate student.

## USE OF LIBRARY SERVICES AND LIBRARIAN SUPPORT

Participants mostly sought information outside of the physical library. Students used the physical library primarily for study space, not necessarily associating it directly with information-seeking activities. One student stated: “The physical library is usually [where] I study, just a physical location.” (Participant 8) The online environment enabled users to seek information not only anywhere, but also anytime. One participant said: “When I am looking for a paper, it is usually either really early in the morning or really late at night. My schedule during the day is a little bit tighter, unfortunately.” (Participant 6)

Although five participants stated they had used the online chat service at least once and had found it useful, the chat was not used frequently. One participant saw it as technical support. Another participant simply said something like this: “I’ve seen it, but I haven’t used it.” (Participant 8)

Twelve participants mentioned the pharmacy librarian in their interview responses. Those who had utilized her services found them to be useful. For example, one participant spoke of their experience contacting the librarian remotely:

I use looking up articles a lot. I don’t physically come here except for studying, but there's been multiple times I’ve emailed Jennifer if I can’t find something and she always knows everything about it, so she can always find it. (Participant 23)

In addition to one-on-one reference services, the course-integrated library instruction provided by the pharmacy librarian also proved helpful. One PharmD described some of the benefits in more detail:

If Jennifer didn’t go through everything with us in Case Studies, and even in some of the other courses that, she talked to us about different sources, I wouldn’t exactly know where to go. I’d probably just mainly use Google Scholar, or Google, or just WebMD. I wouldn’t have known about any other databases out there that has open cases or old studies. (Participant 17)

Those who had previously benefited from the pharmacy librarian used her services more often. A graduate student said: “I ask Jennifer for help all the time.” (Participant 28) A faculty member who stated he had not used the chat service said, “If I really need help, I will ask Jennifer.” (Participant 20) One graduate student mentioned the proximity of the librarian being beneficial: “I like having Jennifer here. Jennifer has an office in the College. That means I have access to the library in my College.” (Participant 25) In addition, when asked about what advice they would give to new students about seeking information, these participants who benefited from the librarian would recommend seeking assistance from the pharmacy librarian.

We noted previously that the participants in this study might have been more inclined to possess positive attitudes toward the library. Notwithstanding this potential bias, no one explicitly expressed discomfort or dissatisfaction with the librarians or library services. They said they feel or would feel comfortable asking a librarian for help and were comfortable using the physical library and library services. However, even if they said they were willing to ask for help, they did not necessarily do so, as they were generally satisfied with their searches. For example:

I haven’t used librarians for help too often. Mostly the extent of it is trying to get something on interlibrary loan. Also, usually I’ve been pretty successful at finding what I need through Google and PubMed. I wouldn’t have a big problem trying to approach a librarian for help if it came to that. It just hasn’t come to that yet. (Participant 6)

Also, one student responded, “I usually try to get it online. There's lots of info online, so I haven’t approached a librarian.” (Participant 8) That student also noted they would feel comfortable asking if necessary. One PharmD student's response implied they were more likely to ask librarians for assistance if they were familiar with the librarians:

I am usually comfortable [asking a librarian for help]. Sometimes if there is somebody I don’t see at the library often, I am kind of shy about it because I don’t know what their scope is and if they know more than I do. But if it is someone I see often in the library, then I get more comfortable about asking how to find sources. (Participant 9)

When asked if they had previously received library instruction or orientation, participants described different levels of experience, and the perceived impact of this experience varied. For example, a PharmD student said, “So the fact that I could look up the toxic levels of vancomycin for my preceptor is because of the training that I got.” (Participant 21) Another PharmD student talked about the impact of early instruction on later information needs:

I remember when I first started, when the librarian came in and explained what resources are available to us. At that time, I did not know the impact of how much that would help me in the future. But as time went on and you start getting into different classes and realizing how much research is important, especially in the critical field, I am glad I was exposed to that. (Participant 18)

On the other hand, one PharmD student made this comment about previous library instruction:

I honestly don’t remember if there was any library instruction. There probably was at some point in my years of undergrad, but I guess it wasn’t good enough, I would say, to leave any lasting impression on me. (Participant 7)

## DISCUSSION

Although the small number of participants who were interviewed may not allow for generalizable results, interview responses showed that individual participants’ information needs and information-seeking behaviors varied. Their information needs differed by their specialized areas and their status within the College of Pharmacy. Their information-seeking behavior was also affected by the fact that faculty and advanced researchers tend to have focused information needs, while students tend to have fewer focused needs, such as completing assignments for coursework. Further, different work settings may affect their behavior. For example, one faculty member in a clinical setting was very conscious about getting information quickly, and the quality of information was very important in this setting. Also, participants’ responses showed that their experience and confidence in information-seeking varied. In general, faculty members seemed to possess more experience and confidence than students.

These results affirm that users mostly seek information outside of the physical library at any time, which indicates that the traditional way of providing information services within a physical library may not be sufficient and thus supports the embedded librarian model. Embedded librarians who are physically located where users study and work may be better positioned to assist users than librarians located within the confines of the library. While chat reference seems like it should be effective in an online environment, users may not use it or use it infrequently. When it was used by study participants, it was for asking relatively simple reference questions. This suggests that a chat service is not yet an effective way to address complex reference transactions. This also supports the embedded librarian model, which enables the librarian to provide customized support for users’ complex information-seeking activities.

Previous research shows that students’ information literacy skills and perceived competencies do not necessarily match [14]. While some of the graduate students in this study struggled with searching at times, overall, most were confident in searching for information. In the current information-rich environment, information seekers are able to find at least some information for their needs and may be satisfied with what they get as a search result. However, they may be missing out on other relevant information. When comprehensiveness is critical, such as when seeking information for patient care, users are more likely to indicate dissatisfaction with their search results. Yet, even in difficult situations, they do not necessarily contact a librarian for assistance. As Haines et al. reports, scientific researchers at a medical school tend not to seek assistance from the library when looking for information [10]. This tendency also supports the embedded librarian model, which aims to assist users in proactive ways rather than waiting for users to seek assistance.

The results of this study indicate that users who were acquainted with the librarian were more willing to seek assistance. Also, users share information, obtain assistance in information-seeking, and receive information among their peers or research groups, so unless they are familiar with the librarian, they turn to their peers first. Thus, librarian assistance should be more effective if a librarian is considered an integral part of these settings. Blake et al. discussed how even in an electronic resource-heavy environment, the physical presence of a librarian is important for encouraging users to seek librarian support and library services more often [5]. Thus, closer proximity to their users increases the effectiveness of embedded librarians.

The results of this study affirm the common practice of initiating searching with known, preferred databases. At the same time, the results highlight the difference in awareness of other database options among respondents. This is in line with Lê et al.'s finding that public health students tend not to be aware of other relevant databases in this area [8]. De Groote, Shultz, and Blecic also discuss this tendency among health sciences faculty [11]. To address such issues, librarian assistance can raise awareness of other database options; however, introducing too many database options may be an issue. Therefore, librarians can provide assistance in helping users choose the right databases for their information needs.

Our participants devised their own preferred way of managing information for research and study. Thus, helping identify relevant information sources for keeping up to date can be another area that embedded librarians can support. Further research in how users receive and manage information should help librarians devise better ways to help in this area. Presenting a few standard approaches may not fit individuals’ behavior and preferences; instead, embedded librarians should provide customized assistance by understanding users’ behavior within their classroom and work environments, a process enhanced by the librarian's co-location within the user community.

## CONCLUSION

Our interviews provide real, descriptive examples of how faculty and students seek and use information at one College of Pharmacy. While this study focused on a specific discipline, it contributes to the existing literature by revealing how embedded librarian services are situated in users’ information-seeking behavior. The results affirm the tendencies revealed in previous studies but also provide details of individual variability, adding another dimension to the study of users’ information-seeking behavior.

Embedded librarians, taking advantage of their proximity to and peer relationship with users, can proactively support those who would normally not seek assistance with finding information. Users may especially benefit from assistance in selecting appropriate resources and locating relevant or sufficient information from search results. Furthermore, gaining a more detailed understanding of users’ behavior in managing information may evolve into more effective library support. Besides general tendencies, understanding individual variations in information-seeking behavior has further implications for improving library services and the embedded librarian model at the UAHSL. The results of this study will feed into a more extensive quantitative study to further examine the impact of embedded librarians in pharmacy education.

## Data Availability

Original interview recordings and transcriptions cannot be made available due to IRB restrictions. Summary of codes and sample transcriptions from the interviews are available in Open Science Framework at https://osf.io/6jdn4.

## SUPPLEMENTAL FILES

**Appendix A:** Interview Questions
