# Supplementary material for: Understanding the information-seeking behavior of pharmacy college faculty, staff, and students: implications for improving embedded librarian services
Source: J Med Libr Assoc. 2021 Apr 1;109(2):286–94. doi: 10.5195/jmla.2021.950 (PMC8270354; doi:10.5195/jmla.2021.950)
Supplement: Supplementary file 1 — Appendix A: Interview Questions [file jmla-109-2-286-s01.docx]

**APPENDIX A: Interview Questions**

Tell us about a recent experience when you needed to locate information for your study or research.

Searching Prompts:

1. Why did you need that information? (What made you think you needed to seek information?)
2. Where did you search and what happened?
3. Why did you search this resource?
4. Do you think of other sources besides those you searched?
5. Why did you search it that way?
6. Was it easy to come up with keywords?
7. Did you have difficulty identifying correct keywords?
8. How did you feel about the results?
   - Did you feel the results were adequate? (What would you like to see happen with this search?)
   - What did you do with this result, or why did you use this result?
9. How do you know when you found what you are looking for?
   - For example, did you continue to search or did you stop searching?
10. During this entire process, is there anything that confused you? What help, if any, you would like?

Conclude Searching Part

1. Can you talk about the most difficult time you had retrieving information?
   - What were some of the challenges you faced?
   - How did you feel about those challenges?

General Research Behavior

1. How often do you retrieve information for your study or research?
   - For courses? For a research project?
   - How do you keep up with your field of research, keep current?
   - Do you have Table of Contents delivered to you via email?
2. How do you keep track of your research?
   - How do you keep track of your search results?
   - Do you use a citation or reference manager?
3. How do you manage references for writing a manuscript?
4. Do you use the reference list at the end of journal articles?
5. Have you ever done a citation search?
   - For example, have you used Scopus or Web of Science?
6. If a new student would ask about searching for articles, what would you tell them?
   - Did you ever use your peers as a resource?

The Library

1. How do you feel about using the library?
2. How do you feel about asking a librarian for help? If not, why not?
3. Did you think about using Chat or “Ask Us” on the library’s website?
4. How comfortable do you feel about contacting a librarian?

Conclusion

1. Were you at another institution in the past 5 years? If so, can you compare library services at that institution to those here?
2. Is there anything you would like us to know about retrieving information through the library?
3. Did you have any library training? How important is training to you?
4. Can you compare your services at your previous library versus the services at the library here?
